# 70 years of fighting the 5 giants – lessons from the front line

**DOI:** 10.1080/17571472.2018.1458448

**Published:** 2018-04-23

**Authors:** John R. Ashton

**Affiliations:** Former President of the United Kingdom Faculty of Public Health and former Chair of the UK Public Health Association, UK

In the year that celebrates the 70th anniversary of the ‘national treasure’ that is the National Health Service, this meeting of the Manchester Medical Society is more than timely. The origins of the NHS are rooted in the fight for social justice which runs not only in Manchester, Liverpool and the NorthWest but across the industrial and commercial north of the country. Next year here in Manchester, we will be commemorating the bi-centenary of the Peterloo Massacre in which 15 people, including one John Ashton, protesting about the poor social conditions and lack of suffrage, were slaughtered in a cavalry charge. This was a defining moment in the development of our democracy, the extension of suffrage and in due course to the extension of public services for the whole population.

On 5 July 1948, Aneurin Bevan, Minister of Health and midwife of the NHS inaugurated its first hospital, The Park, in Davyhulme in Trafford; and today, as we speak, former Health Minister and now elected Mayor of Greater Manchester, Andy Burnham is leading the charge for devolution and integration in partnership with the borough councils of this major conurbation. Along the M62 in Liverpool, William Henry Duncan, the country’s first full-time Medical Officer of Health pioneered a dynamic Victorian town hall based public health movement. His work has in recent years inspired a renaissance of public health going far beyond this region, a renaissance of importance when we come to looking at what the future holds in the next 70 years, not least with the long overdue move to devolution of government in arguably the most centralised country in Europe.

This talk will be in two parts. In the first, I will draw on my recent *Lancet* article of Nicholas Timmins’ formidable review of the first 70 years of the NHS to set the scene and identify some key challenges [1]. These challenges must be addressed if our grandchildren are to be able to benefit from the NHS and the Welfare State in their later years. In the second part, I will lay out my own conclusions based on a lifetime within the NHS and my experiences of trying to ensure that a balanced approach to prevention, treatment and care underpin the pursuit of social justice within a whole systems set of arrangements and characterised by visionary local leadership.

On 1 December 1942, queues stretched from his Majesty’s Stationery Office along High Holborn in London. By lunchtime all copies of Sir William Beveridge’s ground breaking report, Social Insurance and Allied Services [*Cmnd 6404*] had been sold. It was much the same story elsewhere. In Liverpool, my father secured the two volume report that today takes pride of place in my study. Beveridge’s report sits alongside work by others who have guided me in my career: Brian Abel Smith, Douglas Black, Ann Cartwright, Karen Dunnell, Margot Jeffries, Jerry Morris, Richard Titmuss, Peter Townsend and many others associated with the London School of Economics and the London School of Hygiene and Tropical Medicine.

In the introduction to his report, Beveridge enunciated three principles that provided a framework for all that was to follow. First, in supporting the importance of learning from past experience, he spelled out that sectional interests (of doctors), should not be allowed to stand in the way of what was ‘a revolutionary moment in world history....a time for revolutions, not for patching’. Second, he was clear that social insurance – the focus of his terms of reference from Prime Minister Winston Churchill – was only one part of a comprehensive policy of social progress, before going on to declaim his most famous and Bunyonesque passage:

It is one part only of an attack upon five giant evils:

(1) upon the physical Want with which it is directly concerned
(2) upon Disease which often causes that Want and brings other troubles in its train
(3) upon Ignorance which no democracy can afford among its citizens
(4) upon Squalor ...
(5) and upon Idleness which destroys wealth and corrupts men.

Finally, the principle of cooperation between the state and the individual was made explicit.

…..the state in organising security should not stifle incentive, opportunity, responsibility; in establishing a national minimum, it should leave room and encouragement for voluntary action by each individual...

Details of the Beveridge plan were broadcast throughout the day by the BBC in more than 20 languages. Copies of the report were dropped into France and circulated among the troops. Later, they were used by the Workers’ Educational Association in theatres of war as educational material that was subsequently held to have contributed to the election of the Labour government in 1945.

For many people, like my father, an insulin – dependent diabetic on low income with a growing family, the security offered by the prospects of comprehensive social security, including access to health care, was transformative. To academics at the London School of Economics and the London School of Hygiene and Tropical Medicine, the Beveridge Report was a bible for post-war reconstruction and an opportunity to put their intellectual muscle to work for the common good. They were to occupy an influential place in government policy – making that would last some 30 years before the Thatcherite revolution of the 1980s. The subsequent fashion for market – based solutions at all costs in the corridors of Whitehall swept away the consensus of solidarity that had emerged from pre-war hardships and the dark days of the fight against fascism in the Second World War.

By the time Nicholas Timmins published the first edition of *The Five Giants: A Biography of the Welfare State* [1995] that consensus was more than frayed at the edges after 11 years of Thatcherism and the rise of rampant free marketeers. As summed up by Julian le Grand, Richard Titmuss Professor of Social Policy at the London School of Economics, the welfare state had been hit first by an ‘economic hurricane’ and later by ‘an ideological blizzard’*.* The demographic challenge was still to come. The ambition of the war-time generation to build a better future for their children was under attack in a cold climate. In returning to the fray with his new edition of ‘The Five Giants’*,* Timmins has taken on the monumental task of not only bringing his earlier work up to date but also of synthesising some 70 years of social policy in the UK, covering some of the most complex and interlocking areas that between them account for two-thirds of government expenditure.

Timmins describes his *magnum opus* as lying somewhere between ‘1066 and All That’ and Gibbon’s ‘Decline and Fall of the Roman Empire’. In agreeing with this assessment, I would add Salman Rushdie’s ‘Midnight’s Children’. Rushdie’s novel, in tackling the theme of India’s transition from British colonialism to independence through the use of magic realism, touches inadvertently on the surreal nature of many of the Shakespearean plots, subplots, and recurring themes to be found in ‘The Five Giants’*.* Through his device of a spiral treatment of health and social services, education, social security, housing and employment, Timmins provides a perceptive and comprehensive analysis of the British welfare state. I have long held that nobody should be allowed near the National Health Service who has not at least studied Richard Titmuss’ introductory lectures on social administration, written in the post-war period; to that essential bibliography, I would add this second edition by Nick Timmins.

Reading Timmins’ book I felt at times like a dying man with the whole of my life passing before me: hard times and rationing, mum counting every penny, national dried milk and welfare orange, the death of King George VI and the Coronation of Queen Elizabeth II, primary school in Liverpool with more than 40 children in a class in prefabricated huts, school milk and dinners, 6 weeks in Alder Hey Children’s Hospital with suspected meningitis, passing the 11+ examination while my brother failed it and was consigned to an underfunded and undervalued secondary modern school, constant interaction with the NHS for my dad’s diabetes, the discovery of the teenager during the Beatle years, and getting into medical school with a full grant. Then later as a doctor in the 1970s at the tail end of paternalism with ex-colonial administrators and later after the Griffiths Report of a new breed of general managers on huge salaries and flash cars, watching the slow descent of the UK into crisis followed by the rise of monetarism and oscillating nostrums that sapped the energy, without leading to sustainable change in the NHS.

This social history is all documented in Timmins’ truly remarkable book.

He chronicles the battles between different world views, veering between soap opera and epic, with ideology frequently trumping evidence; shallow rhetoric and narrow managerialism in place of authentic leadership, an increasing infatuation with keeping ministers happy in the Westminster bubble, a loss of focus on serving the people and being accountable to them, not least after the abolition of the Regional and District Health Authorities.

Nevertheless, at its heart the flame was kept alive, an enduring ambition to provide social security and freedom from fear for the whole population – for the many, not the few. Nick Timmins lays bare many of the underlying paradoxes, contradictions, and recurring challenges that underpin the muddling through so characteristic of UK Government social policy. He exposes the bankruptcy of politicians who seek short-term advantage at the expense of stability, progress and authentic leadership. Timmins’ blow-by-blow account of former Secretary of State for Health Andrew Lansley’s destructive and fragmentary NHS re-organisation, the orgasm of re-organisation heaped on re-organisation in an ever more frantic crescendo, captures the biggest threat to the NHS since 1948.

Years ago, in another life as a psychiatrist, I had as a patient a young man who was crippled with an obsessive compulsive disorder manifested by elaborate rituals. One day he told me that he had a plan to expunge his problem once and for all by acting out the ultimate in rituals in a local park. A few days later he returned to see me in great distress; he was half way through his ritual when he forgot what to do next. This image came into my mind most vividly at the height of Andrew Lansley’s structuralist madness.

Flawed Victorian notions such as ‘the undeserving and the deserving poor’, the principles of ‘lesser eligibility’ under which it was necessary to be completely destitute before receiving state help, and ‘the workhouse test’ make an unwelcome re-appearance in UK Government social policy dressed in new clothes (the virtual workhouse of Ken Loaches recent shocking film ‘I Daniel Blake’). In an age where we are functioning in a global economy, policies that will only reach part of the population prevent all citizens from reaching their full potential and put the national economy at a disadvantage.

The search for a unified system of tax and benefits continues. Centralisation proceeds apace dressed up as citizen empowerment. Thomas Gradgrind, Charles Dickens unfeeling character in ‘Hard Times*’* is alive and well and living in the Treasury or the Department of Work and Pensions where knowing the price of everything and the value of nothing appears to be a prerequisite for promotion.

But not all politicians are bad guys. Tessa Jowell understood public health as Sarah Wollaston does today, but we are sadly lacking in the kind of leadership which is needed to take us forward to a new era that is fit for purpose. Timmins holds that George Godber and Liam Donaldson have been England’s best Chief Medical Officers. (I would add Donald Acheson) The civil servants in the Department of Health have often been dealt a poor hand in having to deal with contradictory political demands. Having worked closely with Duncan Nichol in our Mersey days I can attest to Duncan’s imagination and support for creative innovation and Simon Stevens is doing his best to unravel the mess that Lansley left behind. At the very beginning Churchill supported Beveridge, whilst the ambivalence of The British Medical Association was worthy of a contortionist.

And in the end many of the questions about how to provide population – based security out of general taxation remain, the ideological and the demographic. Of these, the demographic should be the most straight forward, and the Health Service must adapt to the needs of an increasingly elderly population – after all we have had over 30 years of knowing what was coming! The interdependence of the five giants is just as great today as it ever was. If we are to use our resources to optimal effect for the whole population, we need that interdependency to be understood by those charged with serving the public; we need citizens who understand it too. Timmins book provides a basis for that curriculum and is essential reading for all would-be public sector leaders.

In the second half of this talk, I will describe my conclusions about the agenda for such leaders that can carry us forward for the next 70 years.

So with Nick Timmins and ‘The Five Giants’ in mind we might ask ‘So what is the question that the constant tinkering is supposed to answer?’ The incessant reorganisation and obsession with structure to the detriment of function; the flirtations with privatisation and our fixation on the grossly unfair arrangements that are to be found in the USA where even the Health Maintenance Organisations such as Kaiser Permanente only cover employees; the constant threat of the introduction of regressive health insurance as an alternative to a system funded out of progressive taxation where there is pooling of risk. Meanwhile, we choose to ignore the experience of other countries, such as Finland, which have long since achieved the necessary transformational changes, within modest budgets, to put themselves on a sustainable path.

The starting point is how to optimise the health and well-being of the whole population, equitably, through a system of social security and welfare provision funded out of general taxation. It is daily apparent that essential services, services that make for a productive and healthy population [water and sanitation, energy, mass transit, education, housing and health] are too important to be left to the market, something that Richard Titmuss would have argued passionately 50 years ago. Ironically, we have been here before, as the recent demise of Carillion should remind us. Over 100 years ago the main utilities, including the gasworks and tramways were taken into municipal ownership, as later were coal mines, iron and steel and the railways because as essential services they could not survive without effective public interest oversight. In the fashionable dash to market-based purism that followed in recent decades, the public has become increasingly aware that the most obvious impact of privatisation has typically been the addition of around 10% to bills to satisfy the expectations of shareholders, without adding to the satisfaction of consumers. Health Services are no different, as the huge increase in transaction costs in the NHS over the past 30 years demonstrates. The distress of patients, workers and families that follows market failure from care homes to hospital construction is a price that the public is increasingly not prepared to pay. The burden imposed by the Private Finance Initiative is now seen for what it is: short sighted and obscene. Where were the voices of restraint at the seats of power when those decisions were being made? And those such as Alison Pollock who raised the alarm were ridiculed, vilified and disparaged.

If we ask why the NHS and similar systems in other countries arose in the first place, we find that an ethical impulse was often secondary to the imperatives of Empire and Industry, not to mention the actual survival of elites. In Germany, the ethical argument was made by Neumann in 1847 ‘The State argues that its responsibility is to protect people’s property rights. For most people the only property which they possess is their health; therefore the State has a responsibility to protect people’s health’. So much for the ‘Nanny State’. In fact, it was Bismarck, who, fearing revolution among the young men drawn into the cities by rapid urbanisation in 1848, the year of revolution in Europe, and fearing the spectre of the guillotine from France, implemented reforms in social welfare. In this country the extension of primary education in 1870 was motivated by the realisation that we were falling behind Germany and our European competitors. The consequence of finding that 40% of working class recruits to fight in South Africa in 1899–1902 were unfit for military service led to concerns about ‘how the country could maintain an Imperial Race and contain Germany’ and a comprehensive programme of action was proposed which included:

(1) A continuing anthropometric survey
(2) Registration of still births
(3) Studies of infant mortality
(4) Centres for maternal instruction
(5) Day nurseries
(6) Registration and supervision of working pregnant women
(7) Free school meals and medical inspection
(8) Physical training for children, training in hygiene and mother craft
(9) Prohibition of tobacco sales to children
(10) Education on the evils of drink
(11) Medical on entry to work
(12) Studies of the prevalence and effects of syphilis
(13) Extension of the Health Visiting Service

I don’t know about you but I am ashamed at the state of many of these essential public health services or their equivalent today, despite having had a National Public Health Agency now for six years. These late Victorian and Edwardian measures owe a great deal to Lloyd George who then built on them with his health insurance scheme in 1911, a scheme which resembles that of the American Health Maintenance Organisations which we seem to be so infatuated with despite them mostly being restricted to employees and their families. The great breakthrough with the NHS in 1948, three years after the death of Lloyd George’ was extending cover to the whole population, ‘equal access for equal need, free at the time of use and funded out of general taxation.’ The momentum that lay behind the consensus for the implementation of Beveridge was borne of shared hardship during the economic recession of the 1930s and in the Second World War fronts at home and abroad which transcended social class, although not the front between hospital medicine and general practice, private practice in London and medical practice in the rest of the country. In the event, it has been estimated that GPs doubled their pay in 1948 once bad debts had been taken into account. We have seen time and again how enduring is the public commitment to the NHS as a treasured national institution despite the fanatical determination of those on the political right for privatisation, returning time and time again like space invaders unwilling to learn from experience.

When the NHS was established in 1948, the public health picture was on the cusp of change. Infectious diseases, in particular those of childhood were in rapid decline as a result of improvements in living conditions, nutrition and the advent of comprehensive programmes of immunisation. Maternal and infant mortality rates were still high, certainly in comparison with today and life expectancy was a good deal shorter than now. The remarkable transition to a burden of disease characterised by non-communicable conditions and mental health problems was over 20 years away. However, the manifestations of unmet need were soon making an appearance, notably in relation to dental and optical care. As the pharmaceutical revolution proceeded along with a model of care dominated by hospitals, general practice was neglected and public health, temporarily consigned to the history books. In 1974, the arrangements that had placed the UK at the forefront of public health internationally, led by a Medical Officer of Health from the town hall were laid to rest, marking the high point of this chapter of hospital hegemony. Almost immediately, commentators began to argue the case for a renaissance of public health and for a reorientation of thinking, policy, organisation and practice. In 1976, Birmingham Social Medicine Professor Thomas Mckeown, demonstrated the fallacy that modern medicine had been responsible for the dramatic improvements in mortality rates over the previous 100 plus years; rather, most of the reduction in deaths from tuberculosis, bronchitis, pneumonia, whooping cough, and food and water-borne disease had already occurred before effective immunisation or treatment was available. Progress in these areas had probably had much more to do with smaller family size, improved environmental and housing conditions with advances in hygiene and the improved availability of cheap and safe food. Around the same time researchers such as Ann Cartwright, Peter Townsend and South Wales GP, Julian Tudor Hart began to point to the existence of an ‘Inverse Care Law’ in which the most highly trained doctors were to be found in the most privileged parts of the country and those with the worst health were least able to access high quality health care.

Tragically, over 40 years later and despite much hot air and lip service, we have failed to grasp the nettle, even when provided with the logical narrative and increased funding by Sir Derek Wanless. In 2004, Sir Derek persuaded the then Chancellor of the Exchequer, Gordon Brown to cough up significant extra funding for the NHS on the basis that it should be spent on resourcing a fundamental NHS reorientation to one of full public engagement and an upstream focus on prevention. In the event, the money disappeared into the Private Finance Initiative and a massive increase in clinical salaries. Every time dedicated funding has been identified for public health and prevention it has been diverted into balancing the hospital books. For me, the ‘Choosing Health’ monies, which I never saw, as Director of Public Health, was the epitome of the bad faith which emanated from Richmond House to be implemented at the local level. If I had a penny for every time I was told that we would get round to prevention once we had sorted out the hospitals I would be a rich man. And since the creation of Public Health England, another national body has failed to protect the frontline public health budget, standing by whilst the invidiously placed local authorities have diverted funding away from public health programmes such as family planning and sexual health to balance the books for social care.

Since McKeown published his analysis and others fleshed out our understanding of contemporary patterns of health and disease there has been no excuse for failure to transform our organised efforts and arrangements to optimise population health. This year we will celebrate 40 years of the AlmaAta Declaration made in Kazakhstan in 1978 and which underpinned the World Health Organisation Strategy of Health for all by the year 2000, adopted by the World Health Organisation in 1981. Health Services grounded in a whole population, whole system approach, the reorientation of health care towards primary and community care and upstream to prevention, tackling inequalities in health, full public engagement and partnership working and policies that support health within supportive environments; later this year in Alma Ata [now Almaty] these same principles will be revalidated. Finland is one of those countries that was listening when the Declaration was made. A Primary Care Act was passed which defined once and for all the proportion of capital spending to be dedicated to comprehensive primary and community health care. It included networks of modern community health beds, such as modern cottage hospitals linked to state of the art health centres across the country, a progression of high-quality community mental health facilities and general practitioners playing a key role at the front end of hospitals ensuring appropriate admissions. On a trip to the Finnish county of Karelia, with health service managers from Cumbria and Lancashire, where over 40 years ago Pekka Puska led a pioneering whole county approach to the prevention of coronary deaths, we heard of the systematic work that had skilled up the local population to manage common health conditions for themselves and resulted in a reduction in general practice consultations of between 20 and 30%; the Finnish Government itself was no hostage to commercial interests or allegations of ‘the nanny state’ and had been prepared to use the tools available to it in the form of legislation and taxation to create an environment that really did make ‘healthy choices the easy choices’. On that same trip to a country that in the early 1970s had had a notoriously bad diet, devoid of fresh fruit and vegetables, we visited a factory canteen where the workers were tucking in to an appetising selection of luncheon salads.

While this was going on in Finland and some other countries, in this country we were on a treadmill of structural reorganisation. In my 13 years as Regional Medical Officer, we underwent six – one every two years. Just as I had finished building up a team I had to start all over again. When I left the regional job in 2006 I commented that if I had wished to be a removals worker I would have joined Pickfords. I like to think that, together with colleagues here in the North West, we did make an impact in developing the New Public Health, not least in emulating what we could from Karelia with regard to heart disease and non-communicable disease in the absence of systematic government support; and certainly with teenage pregnancy and abortion, HIV and AIDS against central government opposition or apathy, but with the covert support of Chief Medical Officer, Sir Donald Acheson. How different it looks today with the present incumbents in Richmond House and Public Health England; a central team with little interest in public health and a lack of public health leadership in Public Health England, together with an unwillingness to challenge government, whilst neglecting the withering on the vine of local and regional public health since the transfer back to local authorities, now under the cosh of austerity and the centralisation of expertise and funding into a vanity project, a quasi-public health hospital in Harlow.

The Board of Public Health England is chaired by Sir Derek Myers, the former chief executive of Kensington borough council, where the disastrous Grenfell tower fire occurred last year, the Board itself having been recently recast as an advisory rather than an executive Board. In June last year the Guardian reported that Shelter Chairman Sir Derek and trustee Tony Rice had resigned because of concern over the organisation’s muted response to the Grenfell Tower fire. So what is needed to keep the faith with that noble generation that returned from war and with a bankrupt country delivered a National Health Service that the cynics and those with contrary vested interests said was impossible?

The manifesto to get us back on track and deliver equitable health and well-being to the population within affordable resources has five components which I will briefly outline:

(1) *A clear vision.* This vision is not rocket science and has been around since Thomas Mckeown and the Alma Ata Declaration 40 years ago. It is a vision of a whole system that tackles the 5 Giants, is rooted in public health and strong primary care which is a partnership with the population it serves. Community-Orientated Primary Care with its roots in Peckham in the 1930s, its adolescence in Johannesburg in the 1950s and a range of documented and persuasive experiments since, not least in Jerusalem, South Wales and Finland show the way. The integration of a whole population approach with the skills of epidemiology and public health alongside clinical and social care and a health literate public has to be the future, especially in the digital age. The combination of an anthropologically Place-based approach and Community Orientated Primary Care is a powerful one. The commercial determinants of health and disease must be confronted by both independent voices for public health and governments for whom the population’s health is more important than commercial interests.
(2) *A convincing narrative.* The failure of neither government nor managerial leadership to provide a convincing narrative of the future that we need is a disgrace. Much of it has been implicit but the repeated reorganisations and ill thought through dalliance with the private sector has happened because of the failure to describe the future and take people on a transformational journey. When Sir Derek Wanless published his report I managed to get a personal submission into Prime Minister Blair’s Christmas red box which brought together much of the argument presented in this talk and urged him to take a different path. The complacency of his reply shocked me and we have all lived through what has happened subsequently. In particular, I had suggested that he make use of his Directors of Public Health to argue the logical case for change in order to give the politicians the evidence-based justification to see it through. If anything today Directors of Public Health have been even more marginalised when they could be important allies.
(3) *Authentic leadership.* When Sir Roy Griffiths suggested that Florence Nightingale would be hard pressed to find a satisfactory answer to the question of who was in charge of our hospitals in 1983, the answer was seen to lie with general management. 35 years later it has failed to deliver. Too often, very highly, not to say excessively paid NHS chief executives have failed to provide either leadership or delivery to their communities and have failed to take responsibility when things have gone wrong, often finishing up with national honours *en route*. In part, their recruitment and training is to blame, and the move to greater clinical management is to be welcomed, but we are still producing chief executives and finance directors who are trained in silos with a narrow range of skills when it would be better for them to be trained in regional multi-agency staff colleges that included future leaders from all the health and social care professions, public health, the voluntary sector, the police, the media, academia and political life to list a few. That there can be chief executives from a range of backgrounds including finance who are unable to make sense of health outcome data or be on top of safeguarding and clinical risk management is unacceptable. As Director of Public Health I battled without success over many years to have health items and health data given the same prominence on Monday morning top team agendas as finance data.
(4) *Full public engagement.* The medical model of health services that was inherited from the private sector is not fit for purpose. As George Bernard Shaw put it ‘All professions are conspiracies against the laity’ and medicine is no exception. The nature of a profession is that of putting up one's plate in the high street and seeing those customers who can afford to pay. It is not fundamentally about either a population focus with equity at its heart or empowerment but is about giving away small pieces of expertise in exchange for payment, not wishing to take the bread from its own mouth. The result is the creation of dependency and inappropriate demand that is not in the public interest and results in the deskilling of the workforce. This applies in the relationship between primary and secondary care where innovation of intervention and expertise has tended to remain hospital bound long after it could have been disseminated. Compare the situation with the motor car industry where expensive expertise is to be found in research and development followed by large scale, high quality and economic delivery. We have begun to realise that such a model can apply to long term conditions as well as to surgery. The modern equivalent of the Home Medical Encyclopedia, which my parents depended on before the NHS, is the internet backed up by proper school education in the classroom, by the full range of accessible allied health professionals and by peer led expert patient groups. In public health, there is now mainstream interest in Asset Based Community Development in which individuals and communities are seen as being half full rather than half empty dependent always on professionals coming along to fix them in a state of childlike dependency. It’s time the NHS and its leaders cottoned on.
(5) *Adequate resources*. Resources in the sense of money are usually the first item to be discussed in relation to the National Health Service. However, the way we frame the questions at the moment there can never be enough money. If we turn things on their head and build a system built on healthy public policies, full citizen engagement and a public health grounded clinical system money is the final question and it is about what it takes to make the system work. In a sense Simon Stevens has been trying to pursue this approach with the five Year Forward View, the programme of Vanguards (incidentally a Trotskyist notion of the leaders having all the answers), and the Sustainability and Transformation Plans which whilst well-meaning have slipped into the Richmond House default position of top down planning in nowadays smoke free, darkened rooms.

These five major components of the change we need must be backed up by a proper population evidence base, appropriate capacity and capability, and arrangements, curriculae and institutions that are fit for purpose. In turn, this has significant implications for our understanding of the values and cultures of a wide range of professional groups, their ability to work collegiately across agencies and as equals with members of the public. It would help if politicians had proper induction into the issues along with the officers who they must work with and if there was clear water between the public and private services and self-interest. But the most important element is co-productive, ethical and challenging leadership at all levels and across the whole system.

Here, in Manchester and in the North generally where there is increasing anger at the failure of our centralised national system of government to deliver, not only on the NHS but with tackling all five Giants and beyond Devolution can bring hope. Andy Burnham and Steve Rotherham, the great cities and counties working together as Leaders of Place can use soft and convening power with an outward focus to transform life for millions. They can push the boundaries free from the fossilised processes that hold us back and provide accountable voices for local people; early examples of the momentum for change that is building include homelessness, environmental sustainability, the need for a living wage and the obscenity of very high pay and inequality. Mayor Bloomberg in New York has given us a flavour of the potential of an elected mayor in championing public health and I have personally witnessed the power and influence of 1000 such first citizens committing themselves in cities across Latin America.

I have had the privilege of working with the World Health Organisation Healthy Cities initiative, which now involves over 1400 cities worldwide, for the past 32 years. Next month  February 2018) in Copenhagen there will be a summit of elected city and metropolitan mayors from around the world marking a new phase of political leadership at the city level. The focus will be on six P’s:People, Place and Participation; Peace, Prosperity and the Planet. Beveridge’s five Giants are now a global threat; by working together not just here in Manchester but in concert around the world we can keep the faith with those who gave us the NHS on 5th July 1948.

## Governance

I take personal responsibility for these opinions which are my own.

## Disclosure statement

No potential conflict of interest was reported by the author.
